# Evidence Integration in the Era of Information Flooding—The Advent of the Comprehensive Review

**DOI:** 10.3389/fpubh.2021.763828

**Published:** 2021-09-09

**Authors:** Thomas Hartung

**Affiliations:** Biology, Bloomberg School of Public Health, Johns Hopkins University, Baltimore, MD, United States

**Keywords:** COVID-19, SARS-CoV-2, blood glucose, comprehensive review, artificial intelligence

The article by Logette et al. “*A Machine-Generated View of the Role of Blood Glucose Levels in the Severity of COVID-19”* is an impressive review of this important aspect of the pandemic. It pioneers a new form of evidence integration, which I would like to call a *Comprehensive Review* as it combines evidence-mapping using artificial intelligence based on entity extraction with dedicated narrative reviews, data analysis, modeling and to some extent expert opinion. It is not a systematic review in the sense of evidence-based medicine but this is discussed in the article as well. With 422 citations, 26 figures and 2 tables, tons of supplementary materials and shared datasets, it is Herculean work, but as a result this approach is a one-stop-shop for information on this topic.

The most remarkable scientific response to the COVID-19 pandemic can be nicely illustrated by more than 240,000 scientific articles amassed by April 2021, so just about 15 months. Two hundred forty thousand is the number of full-texts collected and made available through the openly accessible in the CORD-19 database (1)^1^. Probably we have to add many more written not in English and not as easily accessible. Who can read this all and make sense of it? No human being, probably not even a group of human beings. The Logette et al., article shows that at least in part a machine can do for us.

So far, the scientific community employed two principal mechanisms to help digest whatever topic, the narrative and the systematic review. In case of a narrative review, more or less eminent researchers summarize their views often broadly covering an area. The inclusion and exclusion of work is more or less complete, rarely quality-controlled and the integration of findings usually follows the views of the authors. Enormous biases with respect to overrepresentation of own work and those of close collaborators are common. Still, these narrative reviews are very valuable as they condense at least one school of thinking and highlight contributions, which experts in the field have chosen and pre-digested for the reader. They can, however, also represent the roadblocks for novel^1^ and unconventional findings as they tend to focus on the well-established and accepted body of literature, though this depends strongly on the standing and attitude of the authors.

While the narrative review is almost unavoidably biased and opinionated, this aspect is typically disguised, very different from the editorial or commentary type of articles. The author believes strongly that personal opinion has a place in science, especially when backed by facts and references. The series of own food-for-thought articles initiated in 2007 might serve as examples here (2).

Taking a very different approach, a systematic review is aiming to avoid all these biases and non-objective aspects of science. This type of review has evolved out of evidence-based medicine and is typically addressing only one very well-defined question. The Cochrane group (http://www.cochrane.org), previously known as the Cochrane Collaboration has developed the *Cochrane Handbook for Systematic Reviews of Interventions* (3), which is probably the bible of this approach. It lays out a comprehensive search strategy with inclusion and exclusion criteria and typically a strategy for quality assessment and data integration. While hailed for its transparency and objectivity, the generation of systematic reviews has also some shortcomings: the process itself is relatively rigid, and articles fall through the grid if the search strategy was not well-tuned. In addition, they can create tremendous work sieving through thousands of abstracts and later articles. The quality scoring and risk-of-bias analyses are demanding. Typically, the number of qualifying articles in the end are quite few. The challenge of integrating the remaining evidence can be daring, especially when different types of studies are included. However, in almost 50 years, a culture of objective information retrieval has been developed by Cochrane, GRADE^2^ and others, which have produced high-quality reviews that stand up to critical assessments. Their results, which can as well be “we do not have the quality and strength of evidence needed to answer,” are considered the highest quality of evidence. However, we should be aware of the narrow scope and the burden of the process.

More recently, artificial intelligence (A.I.) and especially its subcategories of machine learning and natural language processing have entered this field. They can extract and annotate data, if documents are presented in machine-readable formats, which can be expanded by Optical Character Recognition (OCR), a key example of A.I. use “reading” documents and converting them into text. As many, especially older, publications are only available as pictures not as text files, this is an important technology to compute their contents. This is not error-free but as in so many A.I. applications the increase in data outweighs the quality concerns of the pieces. In fact, the big data approach is exactly opposite to the approach taken in systematic reviews: While the systematic review identifies the best pieces of information by rigorous criteria, big data are defined by the 3 V of Volume (as much as possible), Variety (different types of data) and Velocity (fast and often continuous addition of new data). Scientific literature represents an example of big data and the three characteristics. If we just think of the about 2.5 million articles entering PubMed every year, volume, variety and velocity are quite evident. A.I. support to systematic reviews has come to support and semi-automate these processes.

In this context, the concept of evidence mapping has been developed. An “*evidence map is a systematic search of a broad field to identify gaps in knowledge and/or future research needs that presents results in a user-friendly format, often a visual figure or graph, or a searchable database”* (4). Natural language processing supports the automation of this approach. The article by Logette et al. is a nice example of this.

To some extent, all these forms of integrating evidence together give an overview on a field and help the scientific community to digest the state of an area. Here, Logette et al. are pioneering a new form of data integration, which I would like to call a “Comprehensive Review, which integrates elements of the different forms of evidence integration. They start off with deep learning and natural-language -rocessing applications (entity extraction and linking) to mine and extract structured information from the large number of open access publications of the CORD-19 dataset. Dedicated literature search then substantiates the suggested connections along the pathophysiology of COVID-19. They do not stop in condensing the scientific literature but complement the retrieved facts with data analysis and modeling efforts.

Evidently, the mass extraction of information from scientific literature has some shortcomings. As the authors state: “*The main weakness is that the machine cannot judge the quality of each article, its output is vulnerable to biases within articles and to overrepresentation of potentially erroneous concepts in the literature, and it filters out forefront research that has not yet reached the wider research community.”* It is worthy of note that the evidence-based approach of excluding review articles, deduplication, pre-defined inclusion and exclusion criteria, quality scoring, risk-of-bias analysis, and meta-analysis provides such tools, but cannot really be applied to such massive literature bases. However, the tools are emerging as, for example, our scoping review of quality scoring tools shows (5). Some semi-automation by machine learning might help in the future. Such tools have been recently reviewed (6–8). Notably, SysRev.com offers a free tool for such purposes, which has enabled so far (16 July 2021) 3,984 reviews based on 578,272 documents reviewed with 2,303,661 review answers^3^ to illustrate the enormous uptake of semi-automated systematic reviews in just a few years.

The approach of the Comprehensive Review puts facts first—reminding of the term “factfulness” coined by Hans Rosling and coauthors. This means to separate facts from opinion to the extent possible. Opinion is defined by the Oxford dictionary as “*A view or judgement formed about something, not necessarily based on fact or knowledge.”* The US politician Daniel Patrick “Pat” Moynihan (1927–2003) stated it well “Everyone is entitled to his own opinion, but not his own facts.” I wrote earlier (9) in the context of my field, “*science is based on facts and their discourse. Willingly or unwillingly, facts are mixed with opinion, i.e., views or judgments formed, not necessarily based on fact or knowledge. This is often necessary, where we have controversial facts or no definitive evidence yet, because we need to take decisions or have to prioritize.”* The dichotomy of fact and opinion was already a topic in ancient times: Hippocrates (ca. 460 – 375 BCE) is quoted “*There are in fact two things, science and opinion; the former begets knowledge, the latter ignorance.”* But can we actually avoid opinion in science? Already the Roman emperor Marcus Aurelius Antonius (121 - 180 CE) stated “*Everything we hear is an opinion, not a fact. Everything we see is a perspective, not the truth*.”

For the future of Comprehensive Reviews, I hope that we strengthen both the systematic review character and the opinion part. It would be nice to learn explicitly what the authors opinions are after the enormous evaluation of sometimes controversial and incomplete facts. We will also need to rethink the peer-review process as time and expertise requirements for such Comprehensive Reviews are difficult to be met by individual reviewers; current parallel evaluations of entire papers might be replaced by assigned parts and aspects. Furthermore, the communication and dissemination of such major “capstone” analysis of an area needs to be rethought, which might involve graphical and layman versions, journalistic, social media, and editorial accompanying publications. Ultimately, a journal offering a home, visibility and the necessary support with respect to peer-review and dissemination would leverage Comprehensive Reviews as a mean to survive information flooding. Logette et al. have sent us on a journey to develop the Comprehensive Review as such a form of evidence integration.

## Author Contributions

The author confirms being the sole contributor of this work and has approved it for publication.

## Conflict of Interest

The author declares that the research was conducted in the absence of any commercial or financial relationships that could be construed as a potential conflict of interest.

## Publisher's Note

All claims expressed in this article are solely those of the authors and do not necessarily represent those of their affiliated organizations, or those of the publisher, the editors and the reviewers. Any product that may be evaluated in this article, or claim that may be made by its manufacturer, is not guaranteed or endorsed by the publisher.

## Footnotes

^1^On March 16, 2020, the Allen Institute for AI (AI2), in collaboration with partners at The White House Office of Science and Technology Policy (OSTP), the National Library of Medicine (NLM), the Chan Zuckerburg Initiative (CZI), Microsoft Research, and Kaggle, coordinated by Georgetown University's Center for Security and Emerging Technology (CSET), released the first version of CORD-19. https://www.kaggle.com/allen-institute-for-ai/CORD-19-research-challenge.

^2^ https://www.gradeworkinggroup.org

^3^Please note my conflict of interest as I consult this company of my former PhD student and now Associate Tom Luechtefeld. https://sysrev.com.
